# Influence of Group Size on the Success of Wolves Hunting Bison

**DOI:** 10.1371/journal.pone.0112884

**Published:** 2014-11-12

**Authors:** Daniel R. MacNulty, Aimee Tallian, Daniel R. Stahler, Douglas W. Smith

**Affiliations:** 1 Department of Wildland Resources, Utah State University, Logan, UT, United States of America; 2 Department of Wildland Resources and the Ecology Center, Utah State University, Logan, Utah, United States of America; 3 Yellowstone Wolf Project, Yellowstone Center for Resources, Yellowstone National Park, Mammoth, WY, United States of America; Institut Pluridisciplinaire Hubert Curien, France

## Abstract

An intriguing aspect of social foraging behaviour is that large groups are often no better at capturing prey than are small groups, a pattern that has been attributed to diminished cooperation (i.e., free riding) in large groups. Although this suggests the formation of large groups is unrelated to prey capture, little is known about cooperation in large groups that hunt hard-to-catch prey. Here, we used direct observations of Yellowstone wolves (*Canis lupus*) hunting their most formidable prey, bison (*Bison bison*), to test the hypothesis that large groups are more cooperative when hunting difficult prey. We quantified the relationship between capture success and wolf group size, and compared it to previously reported results for Yellowstone wolves hunting elk (*Cervus elaphus*), a prey that was, on average, 3 times easier to capture than bison. Whereas improvement in elk capture success levelled off at 2–6 wolves, bison capture success levelled off at 9–13 wolves with evidence that it continued to increase beyond 13 wolves. These results are consistent with the hypothesis that hunters in large groups are more cooperative when hunting more formidable prey. Improved ability to capture formidable prey could therefore promote the formation and maintenance of large predator groups, particularly among predators that specialize on such prey.

## Introduction

Enhanced ability to capture prey is a commonly cited benefit of group living in social predators and a classic hypothesis for the evolution of sociality [1]–[4]. Yet, previous research has shown that the benefit of improved hunting success (defined as the likelihood of capturing prey) is generally only realized in small groups. In many group-hunting taxa, ranging from insects to primates, hunting success fails to increase over larger group sizes despite apparent cooperation among hunters [5]–[10]. This nonlinear pattern is well documented in large social carnivores, which have been model organisms in the study of group hunting behavior. Numerous studies show that carnivore hunting success peaks at 2–5 hunters then levels off, or even declines, across larger group sizes [10]–[19]. Although this suggests the formation and maintenance of large groups is unrelated to prey capture, predators that hunt hard-to-catch prey may follow a different pattern.

Theory predicts that the success of predators hunting formidable prey increases across large group sizes [5]. This pattern is attributed to greater cooperation (i.e., increased individual effort) in large groups due to the small chance a solitary hunter will capture such prey by itself. Low solo hunting success promotes cooperation because an additional hunter can improve group hunting success sufficiently to overcome its own costs of hunting (e.g., risk of injury and energetic loss). Conversely, high solo hunting success suppresses cooperation because an additional hunter can do little to improve the outcome and this fails to offset hunting costs. As a result, hunters pursuing relatively easy prey are expected to hold back in large groups, thereby capping further increases in hunting success with group size. A study of wolves (*Canis lupus*) hunting elk (*Cervus elaphus*) supports this prediction: group hunting success leveled off at 4 wolves, which was also the group size beyond which individual effort decreased [10].

Empirical research has yet to establish how group size-specific hunting success (*H_n_*) of large groups varies across prey species that are differentially vulnerable to predation. Behavioral studies of large carnivores, for example, rarely include data on large groups (e.g.,>6 hunters) [14], [16], [17], [19]–[21] and few have measured how *H_n_* varies across prey species. Among those that have, the results were ambiguous [14], [16], [17], [22]. For example, Scheel and Packer [22] found that African lions (*Panthera leo*) were apparently more cooperative when hunting larger, more dangerous prey (e.g., zebra, *Equus burchelli*; buffalo, *Syncerus caffer*), but they observed too few hunts to relate this to changes in *H_n_*. Positive correlation between prey size and group size across the Carnivora [23], [24] is consistent with the prediction that larger groups are more successful hunters of formidable prey. But it is unclear whether this reflects the need to capture large prey to satisfy increased group demands or because larger groups can capture large prey more easily [16], [24].

Here, we use a unique dataset of observations of wolves hunting bison (*Bison bison*) in Yellowstone National Park (YNP) to test the hypothesis that predators in large groups are more cooperative when hunting formidable prey. Bison are the most difficult prey for wolves to kill in North America [25], [26] and in YNP they are 3 times more difficult to kill than elk [27], which are the main year-round prey for Yellowstone wolves [28], [29]. Bison are more difficult to kill than elk because they are larger, more aggressive, and more likely to injure or kill wolves that attack them [30]. As a result, bison require relatively more time to subdue [30], which is characteristic of dangerous prey [31]. Groups of wolves are more likely to attack bison than are solitary wolves [32], but the effect of group size on the ability of wolves to capture bison is unknown. We measured the influence of group size on the probability that wolves attacked and captured bison, and evaluated how it differed relative to comparable results for Yellowstone wolves hunting elk [10]. If large groups are more cooperative when hunting formidable prey, we predicted the success of wolves hunting bison to increase across large group sizes and level off at a group size greater than that of wolves hunting elk.

## Methods

### Ethics statement

We captured and handled wolves following protocols in accord with applicable guidelines from the American Society of Mammalogists [33] and approved by the National Park Service Institutional Animal Care and Use Committee. Yellowstone National Park issued the permit authorizing this study (Study#: YELL-01818; Permit#: YELL-2014-SCI-1818).

### Study area

Yellowstone National Park extends across 891,000 ha of a primarily forested plateau in northwestern Wyoming, USA that ranges from 1500 to 3300 m. Large montane grasslands provide excellent views of wildlife. We observed wolf-bison interactions in the northern portion of YNP, also referred to as the Northern Range (NR; 995 km^2^), and in the central portion of the park (Pelican Valley; 100 km^2^). Low elevations (1500–2000 m) in the NR create the warmest and driest conditions in YNP during winter, providing critical winter range for migratory ungulates including bison and elk [34]. A maintained road runs the length of the NR and provides year-round vehicle access. Pelican Valley is a roadless area at 2500 m elevation. Elk are seasonally present in the valley (May-November) whereas bison persist year-round because they overwinter in geothermal sites [35]. Deep snow around these sites hinders bison movement which generates a higher risk of wolf predation in Pelican Valley than in the NR [27], [36].

### Study population

A total of 41 radio-marked wolves were reintroduced to Yellowstone National Park in 1995–1997 [37]. Wolves observed in this study were either members or descendants of the reintroduced population. In each year following the reintroduction, about 30–50% of the pups born were captured and radio-marked [28]. This study focused mainly on 5 wolf packs: Druid Peak, Geode Creek, Leopold, Mollie's, and Rose Creek. Only the Mollie's pack inhabited Pelican Valley whereas the others occurred in the NR. To facilitate monitoring and research, the Yellowstone Wolf Project maintained radio-collars on at least 2 individuals in each pack [38].

### Behavior Sampling

The methods we used to sample the behavior of wolves hunting bison were the same as those we used previously to sample the behavior of wolves hunting elk [10], [39]. We observed hunting behavior during biannual 30-day follows of 3–5 wolf packs from the ground and fixed-wing aircraft in early (mid-November to mid-December) and late (March) winter and during opportunistic ground and aerial surveys throughout the remainder of the year [28]. Many observations in this study were recorded from the ground in Pelican Valley during a 2–3 week period in March, 1999–2013. Comparable observations were recorded in the NR, 1996–2003. Over half of our observations (60% of 239 wolf-bison encounters) were recorded in Pelican Valley.

**Figure 1:**
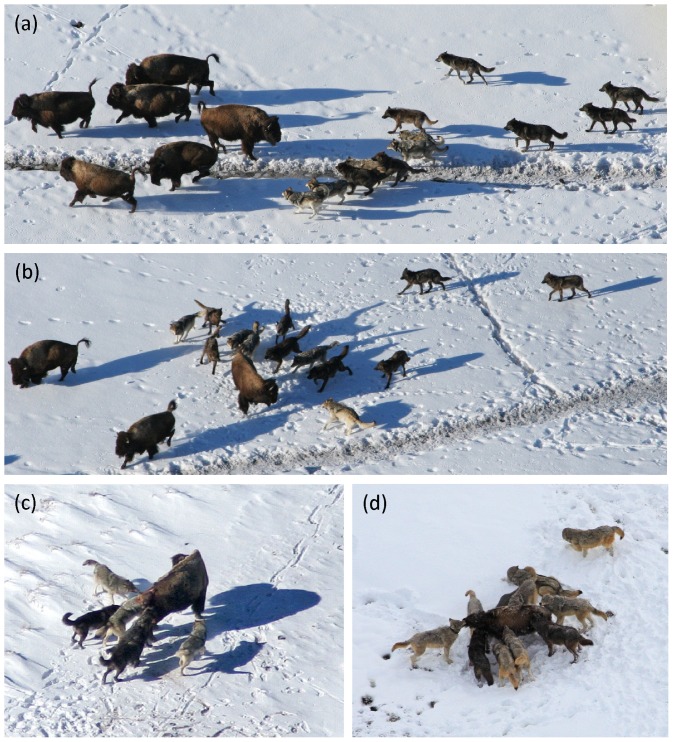
Behavior of wolves hunting bison: (a) approach, (b) attack-individual, (c, d) capture (see **Table 1** for definitions). “Attacking” is the transition from (a) to (b), and “capturing” is the transition from (b) to (c, d). (Photo credit: Daniel Stahler, Douglas Smith).

When wolves encountered bison – defined as at least 1 wolf orienting and moving (walking, trotting or running) toward bison – we followed the progress of the encounter by noting the foraging state (approach, watch, attack-group, attack-individual, capture) of the individual(s) closest to making a kill. We therefore recorded the sequential occurrence of the most escalated state and the number of wolves participating in that state. A wolf was scored as participating in a foraging state if it exhibited the behavioral acts characterizing that particular state (Table 1; Fig. 1). We considered non-participation in a given state as when a wolf was in view but engaged in another foraging state or a non-predatory behavior (e.g., resting). We refer to the number of wolves participating in a foraging state as the “hunting group”. Hunting group size differs from pack size because it pertains to the subset of pack members participating in a hunt. We use “group size” throughout this article to refer to the size of hunting groups. We also recorded the number and age/sex class of bison present at the end of each foraging state. We used body size and horn morphology to identify three age/sex classes: bull, cow, calf [40].

**Table 1:** Ethogram of wolf predatory behavior.

Foraging State	Definition
Approach	Fixating on and traveling toward prey.
Attack-group	Running after a fleeing prey group or lunging at a standing group while glancing about at different group members (i.e., scanning).
Attack-individual	Running after or lunging at a solitary prey or a single member of a prey group while ignoring all other group members.
Capture	Biting and restraining prey.

See [39] for additional details.

We scored group hunting success according to whether wolves completed each of 2 predatory tasks that corresponded to the following 2 behavioral transitions: approach (or watch) → attack-group (or attack-individual)  =  “attacking”; attack-group (or attack-individual) → capture  =  “capturing” (Fig. 1; see also Video S1). Note that capturing was not necessarily killing because bison that were bit and physically restrained by wolves often escaped [39]. A hunting group completed a task, and was therefore “successful”, if the task was performed by at least 1 group member. If not, we considered the group to have “failed” in that task. This scheme generated a binary score for a hunting group in each sequential foraging state.

### Data Analysis

To understand how *H_n_* differs between wolves hunting bison and elk, we followed the same analytical approach that we used previously to estimate *H_n_* of wolves hunting elk [10]. We analyzed how hunting group size influenced the probability that wolves attacked and captured bison based on the binary scores described above. We limited our analyses of capturing to adult bison to control for the effects of prey size on group hunting behavior [5], [10]. Analyses were conducted using generalized linear mixed models (GLMMs) with a binomial error distribution. Such models account for correlation between the multiple observations taken on each pack. Pack identity was fitted as a random intercept to account for the influence of unmeasured pack-related factors on hunting success, including age and size of individuals within packs [41], [42] and differences in prey density between pack territories.

Observations of repeated attempts to perform the same task during the same encounter were also correlated, but these were used in only models of capturing, which fitted encounter identity as a random intercept within pack. Models of attacking included only the first attempt because we were mainly interested in how group size affected the probability of attacking on first encountering bison. All models included a compound symmetric correlation structure, which assumed that all observations within packs and encounters were, on average, equally correlated [43]. Models were estimated with adaptive Gaussian quadrature with parameters estimated from maximum likelihood, and significance of effects determined by an approximate *z*-test.

We used piecewise linear splines to test for nonlinear effects of group size on the probability that wolves attacked and captured bison. Specifically, we tested for a threshold group size beyond which the probability of group hunting success abruptly changed. To determine the presence and position of group-size specific thresholds in attacking and capturing, we evaluated a set of competing GLMMs for each task. Each model set included models with a single knot placed at 2–13 hunters, a model with no knot representing the hypothesis of no threshold in group hunting success, and an intercept-only model representing the null hypothesis that group size had no effect on hunting success. A knot was the join point between two linear splines. We selected knots *a priori* based on the prediction that the success of wolves hunting bison should level off at large group sizes. Our placement of knots is consistent with guidelines for the efficient use of knots [44]–[46]. By definition, knots selected *a priori* are fixed (i.e., not random variables) and are therefore not estimated as parameters in models. We created variables containing a linear spline for group size with the MKSPLINE command in STATA 13.1. The variables were constructed so that the estimated coefficients measure the slopes of the segments before and after a given knot.

To determine if bison herd size and composition affected the relationship between hunting success and group size, we analyzed a subset of observations (N = 92–187 wolf-bison encounters) in which this information was known. First, we evaluated a set of competing GLMMs as above, except that each model also included main effects for bison herd size and composition. The latter was a dummy variable indicating whether a herd was comprised of bulls only or some mixture of bulls, cows, and calves. Second, we tested whether interactions of herd size and composition with wolf group size improved the fit of the top model.

We conducted all analyses in STATA 13.1 and compared GLMMs using information-theoretic statistics [47]. Our scope of inference concerned the population, so we performed model selection using marginal likelihoods. The most parsimonious model was the one with the lowest Akaike Information Criterion (adjusted for small sample, AIC_c_) and smallest ΔAIC_c_. ΔAIC_c_ equals the AIC_c_ for the model of interest minus the smallest AIC_c_ for the set of models being considered. The best model has a ΔAIC_c_ of zero, and models with ΔAIC_c_ <2 are plausibly the best. To assess uncertainty about the best model, we identified models with ΔAIC_c_ <2 as the confidence set of models (analogous to a confidence interval for a mean estimate [47]). We calculated population-averaged fitted values from best-fit GLMMs by deriving marginal expectations of the responses averaged over the random effects but conditional on the observed covariates. We also used likelihood-ratio statistics to test specific hypotheses among nested models, and results were considered significant at *P*<0.05. Means are reported with standard errors unless indicated otherwise.

To determine how *H_n_* differs between wolves hunting bison and elk, we compared our best-fit GLMMs of wolves attacking and capturing bison with our previously reported best-fit GLMMs of wolves attacking and killing elk (Fig. 1a and 1c in [10]). Wolves rarely killed captured bison, but nearly always killed captured elk [39]. Thus, the comparison of capturing with killing is a conservative test given that capturing a bison probably requires fewer wolves than killing it. The transition between attack-group and attack-individual (“selecting”) was rare in wolf-bison encounters [39] and this precluded comparison of the effects of group size on selecting between hunts of bison and elk.

## Results

### Group-size specific success of wolves hunting bison

**Figure 2:**
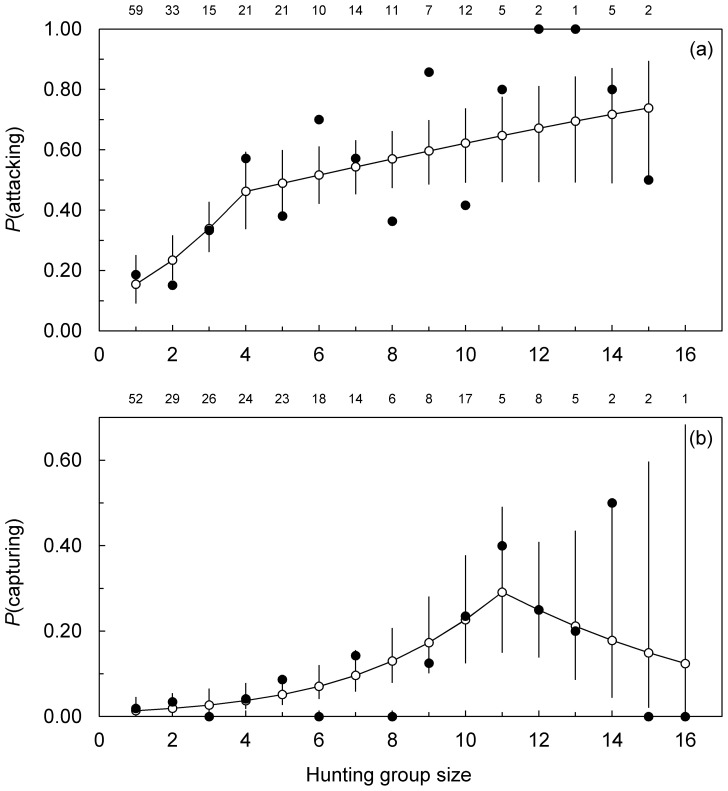
Effects of hunting group size on the probability that wolves attack (a) and capture (b), bison. Open circles are population-averaged fitted values with 95% confidence intervals from the best-fit GLMM models of hunting success (Table S1). The estimated coefficients before and after each breakpoint are: 0.52±0.15 (*P*<0.001) and 0.11±0.07 (*P* = 0.10) (a); 0.34±0.09 (*P*<0.001) and −0.21±0.32 (*P* = 0.50) (b). The number of wolf-bison encounters included in each analysis is: 218 (a) and 106 (b). Filled circles are observed frequencies with sample size indicated above each point. Analyses were performed on the raw binary data and not the illustrated data points, which are provided as a visual aid.

The influence of group size on the success of wolves attacking and capturing bison was not linear (Fig. 2). The top models of attacking and capturing included a linear spline for group size (Table S1), indicating a threshold at which the effect of group size on hunting success suddenly changed. Evidence against a model describing a simple linear relationship between group size and success was reasonably strong for attacking (ΔAIC_c_  = 5.79; Table S1a) but weak for capturing (ΔAIC_c_  = 0.46; Table S1b). The latter suggests that capture success may have increaseed across the largest observed group sizes (11–16 wolves). Yet, the collective fit (summed AIC_c_ weights) of the confidence set of spline models (ΔAIC_c_ <2) was nearly 5 times (AIC_c_ weights  = 0.58/0.12) greater than the linear model, indicating that the effect of group size on capture success was more likely nonlinear than linear. The intercept models fit the data poorly (ΔAIC_c_  = 13.99–32.58), implying that the overall influence of group size on attacking and capturing was strong.

The threshold group size was smaller for attacking than for capturing. The confidence set of spline models for each predatory task (Table S1) indicates the threshold group size was 3–6 wolves for attacking and 9–13 wolves for capturing. The most parsimonious models in the set included thresholds at 4 and 11 wolves for attacking and capturing, respectively (Fig. 2a–b). Beyond each threshold, groups size had no significant effect on success (*P* = 0.10–0.50; Fig. 2). But below these thresholds, each additional wolf improved group success by 67% (odds ratio [OR]  = 1.67±0.25, *P*<0.001) and 40% (OR  = 1.40±0.13, *P*<0.001) in attacking and capturing, respectively. Results were the same for a subset of observations that included data on bison herd size and composition. Moreover, interactions of herd size and composition with wolf group size did not improve fit of top models (attacking: χ^2^_1_ = 0.00–0.63, *P* = 0.23–0.99; capturing: χ^2^_1_ = 0.03–0.96, *P* = 0.33–0.87). Thus, the influence of group size on the success of large groups hunting bison was independent of bison herd size and composition.

### Comparative effects of group size on the success of wolves hunting bison and elk

**Figure 3:**
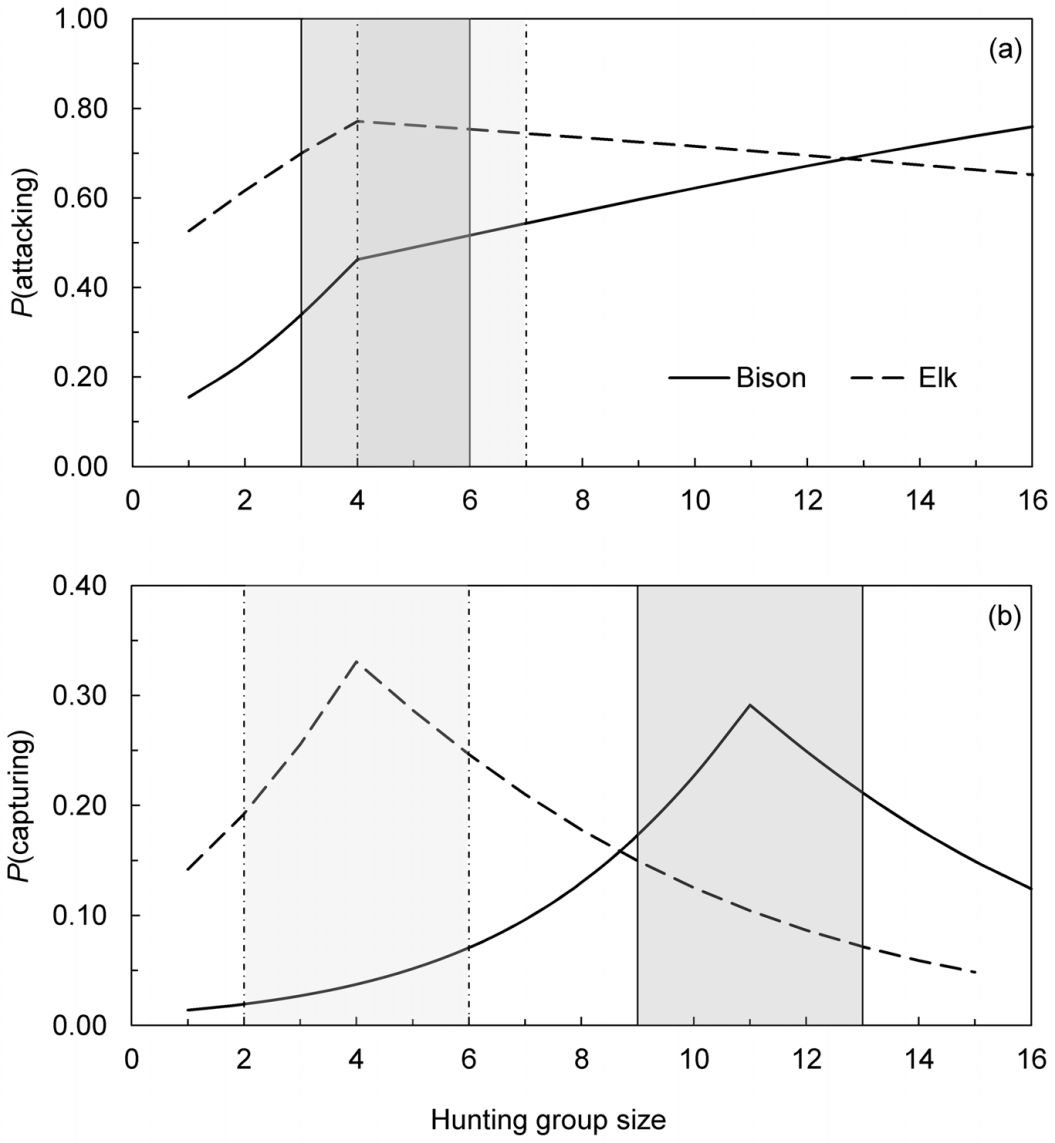
Comparative effects of group size on the success of wolves attacking (a) and capturing (b) bison and elk. Lines are population-averaged fitted values from the best-fit GLMMs of wolves hunting bison (Fig. 2a–b in this study; N = 106–218 wolf-bison encounters) and elk (Fig. 1a, 1c in [10]; N = 235–355 wolf-elk encounters). Slopes were statistically different from zero before each breakpoint (β = 0.34–0.52, SE  = 0.09–0.19, *P*<0.001–0.05) but not after (β =  −0.21–0.11, SE  = 0.05–0.32, *P*>0.10–0.50), indicating that success was effectively constant beyond each breakpoint. Shaded areas (dark  =  bison; light  =  elk) highlight uncertainty about the location of each breakpoint as identified in the confidence set of spline models (ΔAICc <2) for each analysis (Table S1a–b in this study; Table S1a and S1c in [10]). Identical methods were used to collect and analyze data for each species.

Comparing fitted values from our best-fit GLMMs of wolves attacking and capturing bison (Fig. 2a–b) and elk (Fig. 1a, 1c in [10]) revealed a similar influence of group size on the success of wolves hunting these prey insofar as success initially increased with group size then leveled off (Fig. 3). Trends were statistically significant below each threshold group size (*P*<0.001–0.05) but not above (*P*≥0.10–0.50) such that attack and capture success were effectively constant beyond each threshold. Below these thresholds, each additional wolf had a slightly larger effect on the odds of attacking bison (OR  = 1.67) versus elk (OR  = 1.45; Fig. 3a) but a similar effect on the odds of capturing each species (bison: OR  = 1.40; elk: OR  = 1.44; Fig. 3b).

Whereas the threshold group size of wolves attacking bison and elk was the same (4 wolves; Fig. 3a), the threshold group size of wolves capturing bison (11 wolves) was nearly 3 times larger than that of wolves capturing elk (4 wolves; Fig. 3b). This pattern was evident even after accounting for uncertainty about the location of the thresholds (i.e., width of shaded areas in Fig. 3) identified in the confidence set of spline models for each analysis (ΔAICc <2; Table S1a–b in this study; Table S1a and S1c in [10]). Specifically, the range of plausible threshold group sizes was similar when attacking bison (3–6 wolves) and elk (4–7 wolves; Fig. 3a) but higher when capturing bison (9–13 wolves) versus elk (2–6 wolves; Fig. 3b).

Taken together, these results indicate that bison capture success increased across group sizes over which elk capture success was constant (4–11 wolves) and leveled off at a group size larger than that of wolves hunting elk. Given that solo bison capture success (0.01) was 93% less than solo elk capture success (0.14; Fig. 3b), this pattern is consistent with the prediction that large groups are more cooperative when the success of a single hunter is very low.

## Discussion

Our finding that the success of wolves capturing bison increased over large group sizes is unusual. Data from many group-hunting taxa indicate that the benefit of improved hunting success only applies to small groups [5]–[9]. In most carnivore studies, for example, hunting success levels off beyond 2–5 hunters [10]–[19]. Yet, these studies included little or no data on large groups (>6 hunters) hunting difficult-to-catch prey. A notable exception is Creel and Creel [21] who show that the success of wild dogs (*Lycaon pictus*) hunting wildebeest (*Connochaetes taurinus*), a prey they classified as “hard” to capture, increased across large group sizes and leveled off at 12–14 wild dogs, which was comparable to the group size at which the success of wolves hunting bison leveled off (9–13 wolves). Additional studies of large groups hunting formidable prey may therefore reveal that the benefit of improved hunting success is not as limited to small groups as existing studies suggest.

We attribute the increase in bison capture success across large group sizes to enhanced cooperation motivated by the very low capture rate of a single hunter (1%; Fig. 2b). Low solo capture success is expected to foster cooperation because it leaves ample scope for an additional hunter to improve the outcome enough to outweigh its costs of active participation [5]. In support of this prediction, studies of wild dog and African lion have shown that individuals are more likely to participate in a group hunt when the success rate of a single hunter is low [16], [22]. Low solo success was related to larger, more dangerous prey, consistent with our results. And in our previous study of wolves hunting elk, which are >10 times easier than bison for a single wolf to capture (Fig. 3b), we found that wolves in groups with >4 hunters withheld effort, which kept elk capture success constant across large group sizes [10]. Wolves held back at this group size because it was apparently where the costs of hunting exceeded the diminishing improvements in group hunting success with each additional hunter.

In contrast to capture success, the rate at which wolves attacked bison leveled off at small group sizes (3–6 wolves) comparable to that of wolves attacking elk (Fig. 3a). That this reflects reduced cooperation in large groups is consistent with a relatively high rate of solo attack success (15%; Fig. 2a). On the other hand, a positive, albeit statistically weak (*P* = 0.10) trend in attack probability with group size beyond 4 wolves suggests that large groups approaching bison were more cooperative than those approaching elk. Additional data are necessary to resolve this ambiguity.

Another way that formidable prey may increase cooperative hunting behavior in large groups is by affecting group spatial configuration. Simulations from a particle model of group-hunting in wolves suggests that as prey become more dangerous, as measured by a minimum safe distance to prey, the spatial configuration of a group around the prey switches from an unstable, multi-orbit configuration to a stable, single-orbit one [48]. Wolves in the outer orbit of a large group may have less incentive to cooperate than individuals within the inner orbit because they are further from the prey, whereas wolves in a single orbit may more easily contribute to the outcome. Thus, the joint effects of formidable prey on group-spatial dynamics and solo capture success may boost cooperation in large groups. However, our observations of wolves hunting bison suggest that multi-orbit configurations are not exclusive of dangerous prey (Fig. 1b; see also Video S1).

Our evidence that bigger groups were better hunters of larger, more dangerous prey provides rare empirical support for the hypothesis that an advantage of grouping in carnivores is that it increases the diversity and size of prey they can capture [4]. It is well-established that larger groups consume larger prey in Carnivora [21], [23], [24]. But because data on large groups hunting multiple prey species are scarce, it has been difficult to determine whether the correlation between prey size and group size results from greater food requirements of large groups or because large groups can indeed capture large prey more easily [16], [24]. Although our results do not address the relative importance of these two mechanisms, they at least suggest that improved hunting ability is a plausible explanation, despite the tendency of individuals to withhold hunting effort as group size increases [10], [22].

The ability to exploit a wide range of prey is likely a particular advantage in migratory ungulate systems, where the availability of different species is irregular [16]. For example, in Yellowstone's Pelican Valley, where we recorded many wolf-bison interactions, migratory elk were absent in winter (December-April), leaving non-migratory bison as the main prey resource for the resident wolf pack [27], [36]. Correspondence between the mean (± SE) annual size of this pack (10.6±1.1 wolves, 95% CI  = 8.3, 12.9) during the study (1999–2013) and the group size that apparently maximized bison capture success (11 wolves, range  = 9–13; Fig. 2b) implies that this pack is well-adapted to hunting bison. However, this pack also periodically left Pelican Valley in winter to hunt elk in northern Yellowstone, where the size of resident, mainly elk-hunting packs was similar (10.0±0.7 wolves, 95% CI  = 8.7, 11.3). In northern Yellowstone, bison were more often scavenged than killed [29]. Thus, the optimal group size for capturing bison may exceed 11 wolves; a possibility that is supported by our results showing a linear model of the effect of group size on bison capture success fit the data nearly as well as a nonlinear model with a threshold at 11 wolves.

This could explain why wolves in northern and western Yellowstone continue to hunt mainly elk [27], [29], [49] despite decreasing elk availability relative to bison [50]–[52]. On the other hand, wolves in Wood Buffalo National Park, Canada, hunt mainly bison yet live in packs somewhat smaller than those in Yellowstone (8.6±0.7 wolves, 95% CI  = 7.2, 9.9; see Table 27 in [25]). So it seems unlikely that insufficient pack size constrains the ability of Yellowstone wolves to hunt bison. We suspect large wolf packs avoid hunting bison when and where less dangerous prey exist because the profitability (energetic gain/handling time) of bison, discounted for the fitness consequences of injury and probability of injury [31], is relatively low despite improved group hunting success. This highlights how generally invulnerable bison are to wolf predation as well as how the benefit of group hunting for increasing carnivore diet breadth can be contingent on other predator and prey traits that determine the outcome of predator-prey interactions.

Although improved ability to capture formidable prey is not an obvious driver of grouping patterns in Yellowstone wolves, our results demonstrate the potential for such an effect.

This is a significant finding because most empirical studies of group-size specific hunting success imply that the formation and maintenance of large predator groups is unrelated to prey capture. Our study clarifies that the benefit of improved hunting success could favor large groups in populations and species that hunt large, dangerous prey.

## Supporting Information

Table S1 **Model-selection results for GLMM models describing the effects of group size (grp) on the probability that a wolf hunting group attacked (a) and captured (b) bison in Yellowstone National Park, 1996–2013.**
(DOCX)

Video S1 **Group-hunting behavior of wolves attacking and capturing bison in Pelican Valley, Yellowstone National Park, March 2007.**
(WMV)

## References

[1] Alexander RD (1974) The evolution of social behavior. Annual Review of Ecology and Systematics 5: 325–383.

[2] Pulliam H, Caraco T (1984) Living in groups: is there an optimal group size? In: Krebs J, Davies N, editors. Behavioral Ecology: An Evolutionary Approach. 2nd ed. Sunderland, MA: Sinauer. 122–127.

[3] Clark CW, Mangel M (1986) The evolutionary advantages of group foraging. Theoretical Population Biology 30: 45–75.

[4] Kruuk H (1975) Functional aspects of social hunting in carnivores. In: Sibley R, Smith R, editors. Function and Evolution in Behavior. Oxford: Blackwell Scientific Publications. 521–526.

[5] Packer C, Ruttan L (1988) The evolution of cooperative hunting. American Naturalist 132: 159–198.

[6] Boesch C (1994) Cooperative hunting in wild chimpanzees. Animal Behaviour 48: 653–667.

[7] Boesch C, Boesch H (1989) Hunting behavior of wild chimpanzees in the Tai-National Park. American Journal of Physical Anthropology 78: 547–573. 2540662 10.1002/ajpa.1330780410

[8] Rose LM (1997) Vertebrate predation and food-sharing in Cebus and Pan. International Journal of Primatology 18: 727–765.

[9] Kim K, Krafft B, Choe J (2005) Cooperative prey capture by young subsocial spiders I. Fuctional value. Behavioral Ecology and Sociobiology 59: 92–100.

[10] MacNulty DR, Smith DW, Mech LD, Vucetich JA, Packer C (2012) Nonlinear effects of group size on the success of wolves hunting elk. Behavioral Ecology 23: 75–82.

[11] Eaton R (1970) Hunting behavior of the cheetah. Journal of Wildlife Management 34: 56–67.

[12] Kruuk H (1972) The Spotted Hyena. Chicago, IL: The University of Chicago Press.

[13] Schaller G (1972) The Serengeti Lion. Chicago, IL: The University of Chicago Press.

[14] Mills MGL (1985) Related spotted hyaenas forage together but do not cooperate in rearing young. Nature 316: 61–62.

[15] Stander PE (1992) Forgaing dynamics of lions in a semi-arid environment. Canadian Journal of Zoology-Revue Canadienne De Zoologie 70: 8–21.

[16] Fanshawe JH, Fitzgibbon CD (1993) Factors influencing the hunting success of an African wild dog pack. Animal Behaviour 45: 479–490.

[17] Holekamp KE, Smale L, Berg R, Cooper SM (1997) Hunting rates and hunting success in the spotted hyena (*Crocuta crocuta*). Journal of Zoology 242: 1–15.

[18] Funston PJ, Mills MGL, Biggs HC (2001) Factors affecting the hunting success of male and female lions in the Kruger National Park. Journal of Zoology 253: 419–431.

[19] Van Orsdol KG (1984) Foraging behaviour and hunting success of lions in Queen Elizabeth National Park, Uganda. African Journal of Ecology 22: 79–99.

[20] Creel S, Creel NM (1995) Communal hunting and pack size in African wild dogs, (*Lycaon-pictus*). Animal Behaviour 50: 1325–1339.

[21] Creel S, Creel N (2002) The African Wild Dog. Princeton, NJ: Princeton University Press.

[22] Scheel D, Packer C (1991) Group hunting behavior of lions – a search for cooperation. Animal Behaviour 41: 697–709.

[23] Gittleman J (1989) Carnivore group living; comparative trends. In: Gittleman J, editor. Carnivore Behavior, Ecology and Evolution. New York, NY: Cornell University Press. 183–207.

[24] Caro T (1994) Cheetahs of the Serengeti Plains. Chicago, IL: The University of Chicago Press.

[25] Carbyn L, Oosenbrug S, Anions D (1993) Wolves, Bison and the Dynamics Related to the Peace-Athabaska Delta in Canada's Wood Buffalo National Park. Edmonton, Alberta: Canadian Circumpolar Institute.

[26] Mech L, Peterson R (2003) Wolf-prey relations. In: Mech L, Boitani L, editors. Wolves: Behavior, Ecology and Conservation. Chicago, IL: The University of Chicago Press. 131–160.

[27] Smith DW, Mech LD, Meagher M, Clark WE, Jaffe R, et al (2000) Wolf-bison interactions in Yellowstone National Park. Journal of Mammalogy 81: 1128–1135.

[28] Smith DW, Drummer TD, Murphy KM, Guernsey DS, Evans SB (2004) Winter prey selection and estimation of wolf kill rates in Yellowstone National Park, 1995–2000. Journal of Wildlife Management 68: 153–166.

[29] Metz MC, Smith DW, Vucetich JA, Stahler DR, Peterson RO (2012) Seasonal patterns of predation for gray wolves in the multi-prey system of Yellowstone National Park. Journal of Animal Ecology 81: 553–563. 22260633 10.1111/j.1365-2656.2011.01945.x

[30] MacNulty D (2002) The predatory sequence and the influence of injury risk on hunting behavior in the wolf. Saint Paul, MN: The University of Minnesota.

[31] Mukherjee S, Heithaus MR (2013) Dangerous prey and daring predators: a review. Biological Reviews 88: 550–563. 23331494 10.1111/brv.12014

[32] Carbyn LN, Trottier T (1987) Responses of bison on their calving grounds to predation by wolves in Wood Buffalo National Park. Canadian Journal of Zoology-Revue Canadienne De Zoologie 65: 2072–2078.

[33] Sikes RS, Gannon WL, Amer Soc M (2011) Guidelines of the American Society of Mammalogists for the use of wild mammals in research. Journal of Mammalogy 92: 235–253. 10.1093/jmammal/gyw078 PMC5909806 29692469

[34] Houston D (1982) The Northern Yellowstone Elk: Ecology and Management. New York, NY: MacMillan.

[35] MacNulty DR, Plumb GE, Smith DW (2008) Validation of a new video and telemetry system for remotely monitoring wildlife. Journal of Wildlife Management 72: 1834–1844.

[36] MacNulty DR, Varley N, Smith DW (2001) Grizzly bear, *Ursus arctos*, usurps bison calf, *Bison bison*, captured by wolves, *Canis lupus*, in Yellowstone National Park, Wyoming. Canadian Field-Naturalist 115: 495–498.

[37] Bangs EE, Fritts SH (1996) Reintroducing the gray wolf to central Idaho and Yellowstone National Park (vol 24, pg 402, 1996). Wildlife Society Bulletin 24: 780–780.

[38] Stahler DR, MacNulty DR, Wayne RK, vonHoldt B, Smith DW (2013) The adaptive value of morphological, behavioural and life-history traits in reproductive female wolves. Journal of Animal Ecology 82: 222–234. 23043440 10.1111/j.1365-2656.2012.02039.x

[39] MacNulty DR, Mech LD, Smith DW (2007) A proposed ethogram of large-carnivore predatory behavior, exemplified by the wolf. Journal of Mammalogy 88: 595–605.

[40] Fuller W (1959) The horns and teeth as indicators of age in bison. Journal of Wildlife Management 23: 342–344.

[41] MacNulty DR, Smith DW, Mech LD, Eberly LE (2009) Body size and predatory performance in wolves: is bigger better? Journal of Animal Ecology 78: 532–539. 19175444 10.1111/j.1365-2656.2008.01517.x

[42] MacNulty DR, Smith DW, Vucetich JA, Mech LD, Stahler DR, et al (2009) Predatory senescence in ageing wolves. Ecology Letters 12: 1347–1356. 19780789 10.1111/j.1461-0248.2009.01385.x

[43] Weiss R (2005) Modeling Longitudinal Data. New York: Springer.

[44] Wold S (1974) Spline Functions in Data Analysis. Technometrics 16: 1–11.

[45] Eubanks R (1984) Approximate regression models and splines. Communications in Statistics – Theory and Methods 13: 433–484.

[46] Seber G, Wild C (2003) Nonlinear Regression. NewYork: John Wiley and Sons.

[47] Burnham KP, Anderson DR (2002) Model Selection and Multimodal Inference: A Practical Information-Theoretic Approach. New York, New York: Springer.

[48] Escobedo R, Muro C, Spector L, Coppinger R (2014) Group size, individual role differentiation and effectiveness of cooperation in a homogeneous group of hunters. Journal of the Royal Society Interface 11: 20140204. 10.1098/rsif.2014.0204 PMC4006263 24694897

[49] Becker M, Garrott R, White P, Gower C, Bergman E, et al. (2008) Wolf prey selection in an elk-bison system: choice or circumstance? In: Garrott R, White P, Watson F, editors. The Ecology of Large Mammals in Central Yellowstone: Sixteen Years of Integrated Field Studies. New York, NY: Elsevier. 305–337.

[50] Garrott R, White P, Rotella J (2009) The Madison headwaters elk herd: stability in an inherently variable environement. In: Garrott R, White P, Watson F, editors. The Ecology of Large Mammals in Central Yellowstone: Sixteen Years of Integrated Field Studies. New York, NY: Elsevier. 191–216.

[51] White PJ, Wallen RL, Geremia C, Treanor JJ, Blanton DW (2011) Management of Yellowstone bison and brucellosis transmission risk – Implications for conservation and restoration. Biological Conservation 144: 1322–1334.

[52] Cubaynes S, MacNulty D, Stahler D, Quimby K, Smith D, et al (2014) Density dependent intraspecific aggression regulates survival in northern Yellowstone wolves (*Canis lupus*). Journal of Animal Ecology. 10.1111/1365-2656.12238 24749694

