# Supplementary material for: Influence of Group Size on the Success of Wolves Hunting Bison
Source: PLoS One. 2014 Nov 12;9(11):e112884. doi: 10.1371/journal.pone.0112884 (PMC4229308; doi:10.1371/journal.pone.0112884)
Supplement: Table S1 — Model-selection results for GLMM models describing the effects of group size (grp) on the probability that a wolf hunting group attacked (a) and captured (b) bison in Yellowstone National Park, 1996–2013. (DOCX) [file pone.0112884.s001.docx]

**SUPPORTING INFORMATION**

**Table S1.** Model-selection results for GLMM models describing the effects of group size (grp) on the probability that a wolf hunting group attacked (a) and captured (b) bison in Yellowstone National Park, 1996-2013. Variables grp1 and grp2 contain a linear spline for group size at the indicated knot. The intercept and simple-linear models included no knot. Log-likelihood (LL), number of parameters (*K*), AIC_c_, differences in AIC_c_ compared to the best scoring model (ΔAIC_c_), and AIC_c_ weights (*W*) are given for each model. The best model for each predatory task is in boldface.

| Model | | Knot | LL | *K* | AIC_c_ | ∆AIC_c_ | *W* |
| --- | --- | --- | --- | --- | --- | --- | --- |
|  | |  |  |  |  |  |  |
| (a) Attacking | |  |  |  |  |  |  |
|  | intercept | n/a | -144.84 | 2 | 293.74 | 29.36 | 0.00 |
|  | grp | n/a | -130.40 | 3 | 266.91 | 2.53 | 0.07 |
|  | grp1, grp2 | 2 | -130.12 | 4 | 268.43 | 4.05 | 0.03 |
|  | grp1, grp2 | 3 | -128.84 | 4 | 265.86 | 1.48 | 0.11 |
|  | **grp1, grp2** | **4** | **-128.10** | **4** | **264.38** | **0.00** | **0.24** |
|  | grp1, grp2 | 5 | -128.72 | 4 | 265.63 | 1.25 | 0.13 |
|  | grp1, grp2 | 6 | -128.71 | 4 | 265.62 | 1.24 | 0.13 |
|  | grp1, grp2 | 7 | -129.29 | 4 | 266.77 | 2.39 | 0.07 |
|  | grp1, grp2 | 8 | -129.84 | 4 | 267.87 | 3.49 | 0.04 |
|  | grp1, grp2 | 9 | -129.89 | 4 | 267.97 | 3.59 | 0.04 |
|  | grp1, grp2 | 10 | -130.26 | 4 | 268.71 | 4.33 | 0.03 |
|  | grp1, grp2 | 11 | -130.10 | 4 | 268.39 | 4.01 | 0.03 |
|  | grp1, grp2 | 12 | -129.89 | 4 | 267.96 | 3.58 | 0.04 |
|  | grp1, grp2 | 13 | -129.68 | 4 | 267.55 | 3.17 | 0.05 |
|  | |  |  |  |  |  |  |
| (b) Capturing | |  |  |  |  |  |  |
|  | intercept | n/a | -63.92 | 3 | 133.95 | 14.32 | 0.00 |
|  | grp | n/a | -56.02 | 4 | 120.22 | 0.58 | 0.11 |
|  | grp1, grp2 | 2 | -56.02 | 5 | 122.29 | 2.65 | 0.04 |
|  | grp1, grp2 | 3 | -56.02 | **5** | 122.30 | 2.67 | 0.04 |
|  | grp1, grp2 | 4 | -55.98 | 5 | 122.22 | 2.58 | 0.04 |
|  | grp1, grp2 | 5 | -55.91 | 5 | 122.08 | 2.45 | 0.04 |
|  | grp1, grp2 | 6 | -55.94 | 5 | 122.13 | 2.50 | 0.04 |
|  | grp1, grp2 | 7 | -55.78 | 5 | 121.82 | 2.18 | 0.05 |
|  | grp1, grp2 | 8 | -55.71 | 5 | 121.67 | 2.03 | 0.05 |
|  | grp1, grp2 | 9 | -55.46 | 5 | 121.18 | 1.55 | 0.07 |
|  | grp1, grp2 | 10 | -55.08 | 5 | 120.41 | 0.78 | 0.10 |
|  | **grp1, grp2** | **11** | **-54.69** | **5** | 119.64 | **0.00** | **0.15** |
|  | grp1, grp2 | 12 | -54.76 | 5 | 119.77 | 0.14 | 0.14 |
|  | grp1, grp2 | 13 | -54.86 | 5 | 119.98 | 0.35 | 0.12 |

**Video S1.** Group-hunting behavior of wolves attacking and capturing bison in Pelican Valley, Yellowstone National Park, March 2007.
